# Sympathetic Prions

**DOI:** 10.1100/tsw.2001.258

**Published:** 2001-10-13

**Authors:** Markus Glatzel, Adriano Aguzzi

**Affiliations:** Institute of Neuropathology, University Hospital Zürich, Switzerland

**Keywords:** prions, sympathetic nervous system, peripheral nerves, neuroinvasion

## Abstract

Transmissible spongiform encephalopathies are a group of invariably fatal neurodegenerative diseases. The infectious agent is termed prion and is thought to be composed of a modified protein (PrPSc or PrPRES), a protease-resistant conformer of the normal host-encoded membrane glycoprotein, PrPC[1]. Bovine spongiform encephalopathy, scrapie of sheep, and Creutzfeldt-Jakob disease are among the most notable transmissible spongiform encephalopathies. Prions are most efficiently propagated trough intracerebral inoculation, yet the entry point of the infectious agent is often through peripheral sites like the gastrointestinal tract[2,3]. The process by which prions invade the brain is termed neuroinvasion[4]. We and others have speculated that, depending on the amount of infectious agent injected, the injection site, and the strain of prions employed, neuroinvasion can occur either directly via peripheral nerves or first through the lymphoreticular system and then via peripheral nerves[5].

Recent studies demonstrated the importance of $PrP^{C}$ expression on peripheral nerves in order to achieve efficient neuroinvasion[6,7]. Morphological studies looking at the distribution of pathological lesions pointed out the importance of both the vagal nerve and the sympathetic nervous system for neuroinvasion of prions[8,9].

In the July 19 issue of *Neuron*, our laboratory published a study that provides direct evidence that the sympathetic nervous system is essentially involved in the process of neuroinvasion via peripheral nerves. Furthermore, the notion was proposed that sympathetic nerves could represent a prion reservoir.

By altering the innervation of lymphatic organs chemically and immunologically, and by employing transgenic mice we could show the following: 1) mice with a depressed sympathetic nervous system show a significant delay until the development of terminal scrapie disease after peripheral administration of the infectious agent; 2) these mice show a significant reduction of infectious prions and of pathology at the entry site of sympathetic nerves in the central nervous system; 3) mice with an enhanced sympathetic innervation of lymphoreticular organs develop terminal scrapie disease significantly earlier than controls following peripheral administration of infectious prions; 4) these mice show higher titers of the infectious agent in their spleens when compared to controls.

One key interest of our laboratory is to unravel the mechanisms underlying the process of neuroinvasion on a molecular level. This study fills in another piece in this puzzle, and provides support for a model of neuroinvasion that consists of two distinct phases:

- In the first phase the infectious agent colonizes the lymphoreticular system leading to accumulation and replication of prions.
- In the second phase prions gain access to peripheral nerves, including sympathetic nerves, and proceed toward the central nervous system along these structures.

While exceptional progress has been made in this field many details remain to be elucidated. For example, it is not known how the infectious agent gains access to nerve endings, or how prion transport in peripheral nerves is accomplished at the molecular level. These questions are very likely to keep the scientists in the prion research field occupied for the next several years.
